# Aging with health: aging in place strategies of a Portuguese population aged 65 years or older

**DOI:** 10.11606/s1518-8787.2020054001942

**Published:** 2020-11-23

**Authors:** Maria João Bárrios, Rita Marques, Ana Alexandre Fernandes

**Affiliations:** I Universidade de Lisboa Instituto Superior de Ciências Sociais e Políticas Centro de Administração e Políticas Públicas Lisboa Portugal Universidade de Lisboa. Instituto Superior de Ciências Sociais e Políticas. Centro de Administração e Políticas Públicas. Lisboa, Portugal; II Investigador Independente Portugal Investigador Independente. Portugal

**Keywords:** Ageing in Place, Housing, Residence Characteristics, Independent Living

## Abstract

**OBJECTIVE:**

To identify the strategies developed by people in the aging process to keep living in their own home, despite the weaknesses and difficulties they face.

**METHODS:**

The research was carried out based on a survey of population aged 65 years or older in the Portuguese municipality of Portimão. Data were collected by questionnaire, in 2017, and submitted to statistical and content analysis.

**RESULTS:**

Most respondents own their household, where they feel safe and satisfied, and they show awareness and concern about the changes they have to make in their home to stay there. In addition to housing and livability conditions, health, economic resources, social network, and available services play a major role in the community.

**CONCLUSIONS:**

We identified several strategies used by older adults to stay in their households as they age and the ways they mobilize their available resources, as well as constraints of aging in place.

## INTRODUCTION

The population of contemporary mature societies is aging at an accelerated pace. There is an increase in older age groups and a decrease in younger groups. This is the phenomenon of double aging, greatly affecting the level of Social Security Systems. The sharp increase in human longevity is highlighted in this demographic dynamic — representing an achievement of humanity, knowledge, and technology, but also a challenge for society. The fragility associated with the aging of the biological organism and the increase in the number of very old individuals are some of the issues that arise from this. Moreover, responses to various levels of public action are required, as different global, national, and local challenges arise — requiring innovative planning and the creation of adequate policies.

Although the challenges can be analyzed from the perspective of the consequences of individual physiological aging, it is a problem in the field of Public Health. Thus, the collective health of aging people is analyzed by the increase in chronic diseases, situations of comorbidity, and susceptibility to episodes of exacerbation. This framework gains visibility by considering public health as a science of Health Promotion and intervention “based on the knowledge that health is a fundamental resource of the individual, the community and society as a whole.”^1^

Portugal is one of the European countries that requires greater attention to this phenomenon, with older age cohorts representing a considerable part of the population. According to *Eurostat*, in 2018, 21.5% of Portugal's population was aged 65 or older, being the third highest figure in the EU28 (19.7%), after Greece (21.8%) and Italy (22.3%). In the same year, the Portuguese aged 80 or older represented 6.3% of the population, being higher than the EU28 mean (5.6%), only surpassed by Greece (6.9%) and Italy (7%).

A reduction in mortality at all ages originate such values, with a consequent increase in life expectancy. *Eurostat* data for 2017 show that Portugal achieved a high life expectancy at birth, especially for women (78.4 years for men and 84.6 years for women), compared with the EU28 (78.3 years for men and 83.5 years for women). However, it has low values in relation to healthy life expectancy (60.1 years for men and 57 years for women), below the EU28 (63.5 years for men and 64 years for women).

Thus, the increase in life expectancy, especially Portuguese women, has not been followed by good health conditions. Advancing age leads to decreased functionality and increased susceptibility to diseases. Human longevity leads to an increase in chronic diseases–cardiovascular, neoplasms, pulmonary and metabolic–due to sufficient time to develop and risk behaviors, provoking disability and early death^2^. This aspect can be related to progressive loss of functionality (both physical and cognitive) — represented in limitations and disability, resulting in dependence.

Notably, when life extension is not followed by quality of life, individuals require special care and services. Such issues related to aging populations are demonstrated by the figures on life expectancy in good health. Although the family is the main caregiver, demographic aging is related to the very structure and functioning of Portuguese families. According to *Eurostat*, in 2017, with a fertility rate of 1.38 for Portugal and 1.59 for the EU28, families are composed of fewer people, constituting a limited support network and therefore less able to care for their older members. Aspects such as the increase in the number of legal unions, the increase in the number of single-parent and single-person families, as well as the reduction of the elements at the level of the nuclear family are also add. The cohabitation of older parents with their adult children^3^ is becoming less and less frequent, even though the number of families with three generations have been increasing, implying weak generational distances. Note that, Portugal presents a high rate of employed women as well as an increasing number of family nuclei composed exclusively of older adults.

There is an increase in the demand for formal services associated with the decrease in the capacity of responses at the family level. These aspects have justified the growth in residential structures for the older adults (RSOA) and household support services (HSS), as well as the *Rede Nacional de Cuidados Continuados Integrados* (RNCCI — National Network for Integrated Continuing Care). From 2000 to 2015 there was a growth of 66% for RSOA, fulfilling above 90% of the available households^4^. We talk about the hospitalization sought for disability issues and as a consequence of epidemiological transition, increasing chronic and disabling diseases. On the other hand, household support services, despite being the social response that has grown the most in recent years (71% increase between 2000 and 2015), presented the largest decrease in rate of use^4^. Thus, some mismatch between the needs of individuals/families and the services provided at home may be indicated, which could avoid hospitalization.

The challenges in the health and functionality of older individuals can be analyzed by reflecting on the main reasons for hospitalization: (i) increasing physical deterioration; (ii) inability of informal caregivers to provide care; (iii) lack of support in home services^5^. Note that, according to *Eurostat*, in 2011, 12.6% of people aged 85 or older lived in households, and only 14.8% of women and 7.6% of men in these conditions were hospitalized. This data becomes relevant considering that these people desire to remain independent in their households, in comparison to any model of assisted living facility^6^^,^^7^. Most people desire to stay as long as possible in their own households and they believe that the house they are in the moment is where they will live their entire life^8^. This occurs because they want to remain in a familiar environment, in their home and community^9^.

As an alternative to early and/or unwanted hospitalization, the *aging in place* (AiP) paradigm begins to take form. In essence, it shapes the assumptions that value that people age and stay as long as possible in their own *habitat.* Social policies aimed to aging consider the promotion of conditions and opportunities to people stay at home and age where they wish as fundamental. It is necessary that households follow the aging process of the person and that they are able to adapt their life and conditions of habitability in order to achieve these goals. In fact, aging at home and at your neighborhood implies comprehensive awareness and preparation, especially at two levels: (i) the adaptation of the ecosystem of populations throughout life and (II) individual strategies.

Considering these two levels, this study aimed to know the strategies people use to stay in their homes as long as possible and how they mobilize the available resources to do so.

### Aging in place (AiP), an alternative paradigm for aging?

Over the past decades we have seen increasingly positive paradigms related to public policies and the society in general, capable of changing the way older people are understood and the aging process. The concept of AiP is an example of these positive orientations, which means living at home and in the community, safely and independently, as one grows old^10^. This concept arises as support for the creation of conditions so that people can stay in their own *habitat* for as long as possible as they age, even if they suffer from some disease or decline, whether functional or cognitive. In fact, this paradigm idealizes the household and the surrounding community as privileged places to age^11^.

According to Pynoos, Caraviello, and Cicero^12^, AiP is an emerging policy that focuses primarily on understanding changes throughout aging and the environment in which the person is inserted. For Martin, Santinha, Rito, and Almeida^13^, AiP focuses on understanding the changes associated with aging and the surrounding environment, prioritizing the maintenance of the person either in their own housing or in other structured situations in the community.

Notably, those who age in their communities, remain independent and autonomous for longer and they keep their social support networks active. Hospitalization may cause them greater and/or faster loss of autonomy because daily routine and activities (DRL) — as cooking and cleaning the house — will disappear. Tomasini and Alves^14^ state that institutional environments require very little from older people, and when they leave their homes, they often lose their social relations, weakening social ties. In this argumentative line, it is also known that older people associate AiP with autonomy and independence, regarding the ability to carry out their choices, acceding the services they understand, enjoying social relations and feeling safe^9^.

This perspective of AiP as beyond-housing aspects is related to the literature and interest of environmental Gerontology, to the extent that the entire ecosystem, especially the neighborhood where one ages, affects people's health, being an environment where older people may have greater sensitivity, due to biological changes or the long age of the housings^15^^,^^16^.

According to the OCDE^17^, AiP comprises four dimensions: (I) housing, where it is necessary to consider the adaptation, accessibility, maintenance, and housing alternatives; (ii) integrated services, namely integrated care, HSS and RNCCI; (iii) transport, embracing the way people move, which can be a facilitating or limiting link; and (iv) the neighborhood/community in which the person is involved. Similarly, the World Health Organization (WHO) considers that the success of a AiP stems from a complex approach of several levels of intervention, highlighting: people, places, products, personalized services, and social support policies^10^. Also, we should consider the dimensions of AiP identified by Iecovich^9^, that are interrelated: physical (place), social (personal relationships), emotional and psychological (related to the feeling of belonging and attachment to a place) and cultural (relative to values and beliefs that people attribute to places). In fact, in addition to being considered advantageous, AiP is valued by older people regarding a sense of attachment or connection, and security and familiarity with households and communities, as well as a sense of identity, both for independence and autonomy, and for relationships and care in the places where they live^15^.

This concept can be analyzed according to the ecological model, which assumes that the standards of well-being, health, and functioning of the person are interconnected to influences of biological, behavioral, social, physical, and environmental resources that surround the persons, their families, and community^13^. The ecological model, by illustrating the physiological, behavioral, social, and environmental changes that occur both at individual and community level, as well as the relationships between these changes that play their role throughout the aging process^18^, will ease the identification of gaps, priorities, needs, and interventions.

Wang et al.^19^ consider that effective means of supporting older adults are extremely significant in public health, even able to prolong an independent life. The exercise of this archetype, supported by health promotion and the prevention of addiction throughout the life cycle, can produce significant effects. Such effects are due to the declines (physical and cognitive) that can occur with aging constitute barriers to an independent lifestyle, and encouraging behavioral changes can reduce health-related spending for older people^20^.

## METHODS

Considering the possibilities of AiP, but also the challenges and dimensions for aging people, this study design aimed to identify the strategies developed by people in the process of aging to keep living in their own home, despite the frailties and difficulties they face.

Portimão municipality was chosen to conduct the operationalization this research objective. According to INE – Portugal, in this municipality from Algarve, in 2018, 19.8% of the population was aged 65 or older, being relatively lower than the 21.7% of Portugal as a whole. Still, it is a fairly old municipality, with 120.3 older adults per 100 young people and 31.1 older adults per 100 people on working age.

In the data collection conducted in 2017, the semi-structured interview was selected as technique, supported by a questionnaire built for this purpose, containing open-ended (short) and close-ended questions, considering the significance of characterizing the respondents based on their health and living conditions, identifying the daily needs, strategies used, and aspirations and anxieties when thinking about the future.

The sample was composed of residents of the Portimão municipality, aged 65 and older, non-hospitalized, and living in different social and health conditions. The respondents were selected for convenience, that is, people who made themselves available to collaborate with the research, by a snowball methodology, seeking to bring together both genders. In total, 50 interviews were conducted with people who met the inclusion criteria.

The data collected from the interviews were treated in two different ways. Regarding the close-ended questions (quantitative approach), the statistical analysis program IBM SPSS Statistics 25 was used, allowing uni and bivariate statistical analysis. Open-ended questions (qualitative approach) were submitted to the content analysis technique, enabling the categories construction.

## RESULTS AND DISCUSSION

### Sample Characterization

The sample gathered 50 people, aged between 65 and 91 years, distributed in several age groups: 46% of the sample aged between 65 and 75 years; 42%, aged between 75 and 84 years; and 12%, aged 85 years or older. Regarding gender, the sample achieved a good balance, with 25 women and 25 men.

The most prevalent marital status was the married one, corresponding to 62% of the participants, being 20% widowed, 14% divorced, and 4% single. The overwhelming majority of the sample was retired (92%) and did not maintain any activity.

Regarding the schooling level of the sample, most participants were poorly educated — 50% completed the first cycle (up to fourth grade), 10% the second cycle (up to sixth grade), and 4% the third cycle (ninth grade) — only 8% completed the high education, and 12% presented higher education. Note that, 16% of the respondents did not complete any level of education and that the individuals with lower education level are mainly women (68% of the women completed only the first cycle, and 20% did not study at all). This low schooling level agrees with other studies in Portugal with people aged 65 and older^21^^–^^23^, and it must be considered due to the need for information and implementation of resources necessary for healthy aging and AiP.

### Housing Conditions

**Figure 1:**
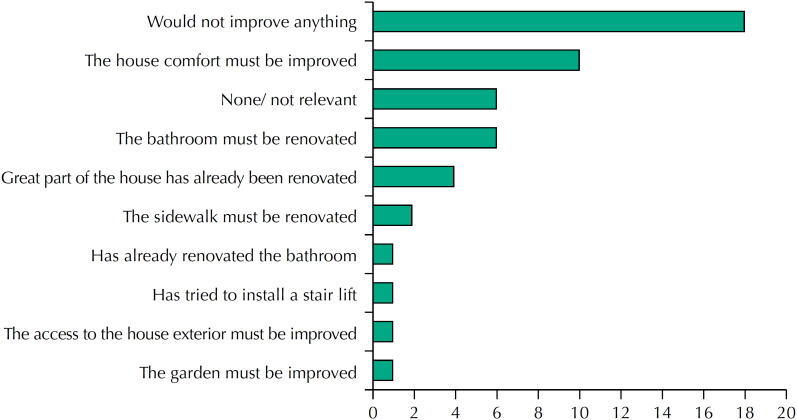
Household renovation.

Most participants have their own home (92%), half live in a dwelling, most (56%) have a garden or yard and 82% have more than four rooms. Most respondents are satisfied with their households regarding safety (74% satisfied, 18% very satisfied, and 2% completely satisfied) and make a positive assessment of its conservation state (60% good and 12% very good). Considering that the household can expose people to several health risks, it is essential to reflect the conditions and improvements of the houses where one ages for the analysis of Population Health. According to the WHO^24^, improving household conditions can save lives, prevent disease, improve quality of life, reduce poverty, and mitigate the effects of climate change. Regarding the modifications they would make to the household, comfort-related, such as painting and decoration, and accessibility, such as adequate bathrooms, for example, stand out (Figure 1). This scenario meets the OCDE^25^, stating that, in Portugal, living in households with satisfactory conditions is one of the most important aspects of people's lives. Note that, most respondents (96%), especially men (100%), consider that the house embraces the necessary conditions to live safely for more years, although they highlight the need for small interventions regarding the state of conservation.

**Figure 2:**
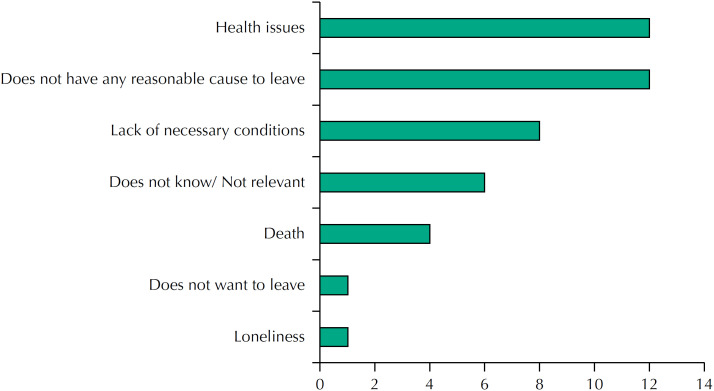
What would cause you to leave your household?

These results show the desire to stay as long as possible in the household itself, placing hospitalization as the last option. Only one case (a man) revealed the opposite option. However, these people are aware of several scenarios that might occur, and if they occur, they may force these people to leave their dwelling. Health problems, lack of conditions and safety of the housing itself and, as a maximum exponent, death were mentioned (Figure 2).

**Figure 3:**
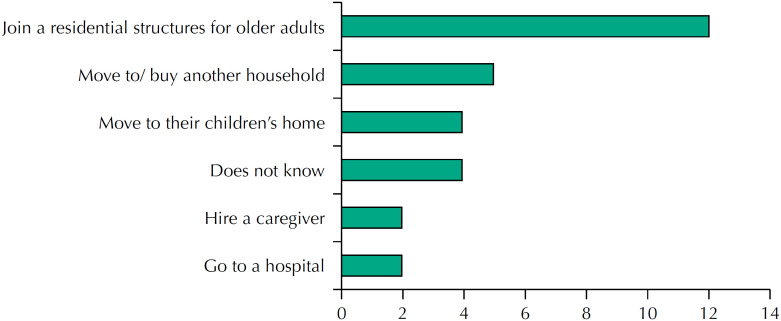
Options.

When they were asked what options they would take if they had to leave home, 24% (12 individuals) would resort to a collective residence (Figure 3).

**Figure 4:**
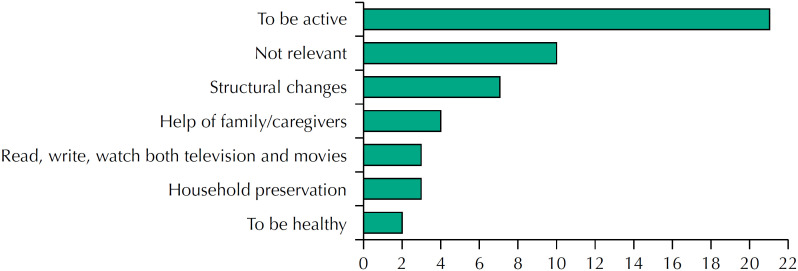
Strategies to stay at their household.

About the strategies they use to stay longer in their own household, the answers are: be active (42%, 21 respondents), make changes in the household structure (14%, 7), or they receive help from family/employees (6%, 4) (Figure 4). Although inactive, the interviewees revealed knowledge of the significance of healthy lifestyles, housing conditions, and the provision of care, whether informal or formal.

### Conditions and Management of Health/Disease

Advancing age imposes a trend of decline in health. In this determinant of the aging process, many respondents describe their health as moderate (46%), 30% as good and 16% as bad, highlighting that older people make a less positive assessment (50% of individuals aged 85 or older consider their health as bad). These less favorable results are the result of diagnosed diseases and limitations. The most common pathologies were back problems, hypertension, diabetes, arthrosis, respiratory diseases and heart conditions. These data corroborate another study^26^, concluding that the most frequent diseases in the Portuguese population aged 50 years or older consist of: diabetes, hypertension, rheumatic disease, osteoporosis, renal calculi, and depression, highlighting arterial hypertension as the most common chronic condition.

Despite the conditions and limitations, as well as the vast range of medicine respondents claim to intake, most participants have never felt that age prevented them from doing the activities they enjoy the most and those daily activities. However, with aging, especially in the 85+ group, the scenario reverses, and people usually consider that age often prevents them from performing activities. Note that, the respondents referred to the vulnerability and weakness of the state of health that advancing age entails, and not to the effects of age by itself.

Regarding the dimension of health service in AiP, none of the respondents resort to any type of home service. In turn, the values represented in the Social Chart^4^ referring to the municipality of Portimão reveal that the social response of HSS has a capacity for 295 users, being used by 179. Therefore, we noticed that respondents revealed concerns around the provision of social and health care in the future. However, the relationship between needs and the diversity of existing/available services requires further exploration. Nevertheless, these results agree with the literature regarding AiP for older people, emphasizing that a successful AiP requires the provision of Health Services at home^27^.

### Means of Transport Used

Regarding the transport dimension, it was found that their use varies according to age. In the 65-74 years group, the most used type of transport is the car; in the 75-84 years group, it is public transport; and in the 85+ years group, the results are divided between walking and public transport. Regarding gender, men usually use their own car, and women, public transport. In this field of intervention, several changes are suggested in order to improve the transport network, as well as the people's interests, that is, straight and more frequent routes, also indicating the existence of transport on weekends. The fact that the transport network does not correspond to the people's needs can result in isolation and less social interactions, if people cannot locomote throughout the city, often not leaving their households. These considerations are increasingly highlighted by the AiP literature, emphasizing that it is necessary to consider not only housing options, but also transportation, which connects people with recreational opportunities, social interaction, cultural involvement, and permanent education^15^^,^^28^.

### Characteristics of the Community where One Ages

Most respondents feel safe and feel that the community provides the resources they need for day-to-day living. However, they highlight some needs, such as wards, homes, public spaces, and a butcher's shop, for example. The simple fact that people feel safe in their community influences the conditions of independence, physical health, social integration, and emotional well-being, because they move around without fear^29^. All respondents mentioned a range of activities that make up their daily lives, and, regarding aspirations and anxieties, the participants expressed the desire to remain in their own household and in the community, leaving hospitalization as the last resource and implying that they do not wish to move, even if it is the only option for some. We were also able to ascertain a health-related concern associated with diseases, physical disability, and apprehension regarding loneliness.

The sample considered that, to have a better quality of life, they need to “maintain the same life,” performing the same activities throughout life, having the ability to perform AVD and AIVD. This set of responses refers to three areas of intervention necessary for establishment of a AiP: (I) strategies related to housing; (ii) adequacy of health services; and (iii) participation and active involvement throughout life.

## CONCLUSIONS

The research identified some concerns and strategies that people develop to stay in their homes as long as possible, throughout the aging process, as well as how they mobilize the available. The conditions and needs of health throughout life are prominent both regarding the respondents’ perceptions, and the literature that supports the challenges and requirements of the practice of AiP. At the heart of the decision and possibility of AiP are housing and habitability conditions. Other variables are also prominent in the research, such as economic and health conditions and the social support network. The strategic documents of WHO^24^ already warn the interconnection of all these dimensions, relating household characteristics with the risk of diseases.

However, the full completion of the AiP paradigm supports a wide range of needs, which can be understood as constraints. In the studied sample, we especially address the economic problem, to the extent that most respondents revealed low literacy and low economic resources, and that fact may limit the necessary changes in their housing, for example. These issues intersect with AiP and public health as considering that there is a growing concern about the quality of the older adults’ households, in terms of insulation, heating/cooling, size, and design/accessibility^15^^,^^30^.

Another dimension of the AiP constraints concerns services. Although the analysis of home care is less expensive than institutional care^31^, the availability of household support services in the Portuguese context is reduced, and the production of these goods — which tend to be expensive by the existing market — creates inequalities, excluding the older population and with low incomes.

These conclusions also lead us to reflect on the inequality of opportunities for aging in the community, that is: Can aging in the community represent a poor quality of life for the population with low income? By contrast, will people with better economic conditions have better ability to adapt and stay in their homes?

The age and limitations imposed by it do not necessarily have to force or to decree one to move to a collective residence, especially if individuals do not yet suffer from problems capable of preventing life in community, whether they are physical, psychological or social. In this sense, community and academic projects emerge, creating strategies for this people may stay and age in their homes. In the Fonseca's^32^ report, 81 initiatives are shared, characterized by categories of intervention: support for caregivers; combating isolation; gerontechnologies and research; innovation in home support; innovation in day care; intervention in community life; leisure, physical activity, and lifelong learning; improvement of housing conditions; health resources, stimulation, nutrition, and psychological monitoring; safety, mobility, and well-being.

However, despite the desire shown in the research to age in their *habitat*, it is important to remember that the interviewees do not underestimate hospitalization and their options, when necessary. Such options are scenarios of frailty and serious illness, with necessary full-time health care, or situations in which the household no longer suits the limitations of functionality. Still, and even in these situations, respondents consider hospitalization as a last resort, giving priority to family members, namely their children, stating that they would resort to informal care services for daily help.

The strategies and needs indicated by the interviewees highlight interventions at several levels, composing the central conclusion of this research. The first level concerns housing. Although respondents are satisfied with their homes, they recognize that in the future it will be necessary to adapt the household in order to keep them safe and allow them to age in their households. A second level is the provision of integrated services (social and health), either by home support or under the configuration of permanent service, that is, a caregiver present in the person's daily life. The third level of intervention concerns the adequacy of community services, addressing the characteristics and functioning of public transport, adjusting them to the interests of the aging population. This occurs because, despite feeling safe and positively mobilizing the resources they have at their disposal, people who live farther from the downtown feel a lack of transport options.
