# Erratum

**DOI:** 10.11606/S01518-8787.2016055001942err

**Published:** 2021-04-23

No artigo **“Envelhecer com saúde: estratégias de *ageing in place* de uma população portuguesa com 65 anos ou mais”**, com número de DOI: https://doi.org/10.11606/s1518-8787.2020054001942, publicado no periódico Revista de Saúde Pública. 2020; 54:129, na página 11, após as Referências e antes da Contribuição dos Autores, faltou mencionar a seguinte informação:

**Financiamento:** Este trabalho foi financiado por fundos nacionais através da FCT – Fundação para a Ciência e a Tecnologia, I.P., no âmbito do projeto UIDP/00713/2020.

In the article **“Aging with health: aging in place strategies of a Portuguese population aged 65 years or older”**, DOI https://doi.org/10.11606/s1518-8787.2020054001942, published on the Revista de Saúde Pública. 2020; 54:129, on page 11, after the References and before the Authors' Contribution, it should be mentioned the following information:

**Funding:** This study was financed by national funds through the FCT – *Fundação para a Ciência e a Tecnologia*, I.P., under the project UIDP/00713/2020.

