# Supplementary material for: Aging with health: aging in place strategies of a Portuguese population aged 65 years or older
Source: Rev Saude Publica. 2020 Nov 23;54:129. doi: 10.11606/s1518-8787.2020054001942 (PMC7688258; doi:10.11606/s1518-8787.2020054001942)
Supplement: Translation

# Translation: Envelhecer com saúde: estratégias de *ageing in place* de uma população portuguesa com 65 anos ou mais

**Authors:** Maria João Bárrios, Rita Marques, Ana Alexandre Fernandes

**Affiliations:** I Lisboa Portugal Universidade de Lisboa. Instituto Superior de Ciências Sociais e Políticas. Centro de Administração e Políticas Públicas. Lisboa, Portugal; II Portugal Investigador Independente. Portugal

**Keywords:** Envelhecimento na Comunidade, Habitação, Características de Residência, Vida Independente

## Abstract

**OBJETIVO:**

Identificar as estratégias desenvolvidas por pessoas em processo de envelhecimento para se manter residindo na própria casa, apesar das fragilidades e das dificuldades com que deparam.

**MÉTODOS:**

A pesquisa foi realizada a partir de um inquérito a uma população com 65 anos ou mais no município português de Portimão. Os dados foram recolhidos por questionário, em 2017, e submetidos a análise estatística e análise de conteúdo.

**RESULTADOS:**

A maioria dos inquiridos tem habitação própria, onde se sente segura e satisfeita, e revela ter consciência e preocupação em relação às alterações que deve executar em sua casa para ali poder permanecer. Além das condições habitacionais e de habitabilidade, as condições de saúde, os recursos económicos, a rede social e os serviços disponíveis assumem um papel preponderante no envelhecimento na comunidade.

**CONCLUSÕES:**

Foi possível identificar uma diversidade nas estratégias utilizadas para permanecerem em suas casas à medida que envelhecem e uma multiplicidade de formas como mobilizaram os recursos que têm à disposição, bem como constrições do *ageing in place*.

## INTRODUÇÃO

A população das sociedades maduras contemporâneas está envelhecendo a um ritmo cada vez mais acelerado. Trata-se de um aumento dos grupos etários mais velhos e de uma diminuição dos grupos mais jovens. É o fenômeno do duplo envelhecimento, com impactos muito significativos no nível dos sistemas instituídos de segurança social. Nesta dinâmica demográfica, realça-se o crescimento acentuado da longevidade humana, que representa uma conquista da humanidade, do conhecimento e da tecnologia, mas também um desafio para a sociedade. A fragilidade associada ao envelhecimento do organismo biológico de que somos dotados e o aumento do número de indivíduos muito velhos são alguns dos problemas que daqui decorrem. Acresce que são exigidas respostas a vários níveis de ação pública, ao se constituírem diferentes desafios globais, nacionais e locais, que requerem um planeamento inovador e a criação de políticas que respondam de forma adequada.

Ainda que os desafios possam ser analisados na perspectiva das consequências do envelhecimento fisiológico individual, trata-se de uma problemática no âmbito da saúde pública. A saúde coletiva das pessoas que envelhecem é, assim, analisada pelo aumento das doenças crônicas, situações de comorbilidade e suscetibilidade a episódios de agudização. Este enquadramento ganha visibilidade ao considerarmos a saúde pública como ciência de promoção da saúde e intervenção com “com base no conhecimento de que a saúde é um recurso fundamental do indivíduo, da comunidade e da sociedade como um todo”^1^.

Portugal é um dos países europeus onde este fenômeno exige maior atenção, com as coortes etárias mais velhas representando uma proporção considerável da população. De acordo com o *Eurostat*, em 2018, 21,5% da população em Portugal tinha 65 ou mais anos, sendo o terceiro valor mais elevado da UE28 (19,7%), depois da Grécia (21,8%) e da Itália (22,3%). No mesmo ano, os portugueses com 80 ou mais anos representavam já 6,3% da população, sendo superior à média da UE28 (5,6%), novamente apenas superada pela Grécia (6,9%) e pela Itália (7%).

Estes valores devem-se a uma redução da mortalidade em todas as idades, com consequente aumento da esperança de vida. Os dados do *Eurostat* de 2017 mostram que Portugal atingiu uma esperança de vida à nascença elevada, sobretudo para mulheres (78,4 anos para homens e 84,6 anos para mulheres), em comparação com a UE28 (78,3 anos para homens e 83,5 anos para mulheres). No entanto, apresenta valores baixos em relação à esperança de vida em boa saúde (60,1 anos para homens e 57 anos para mulheres), abaixo da UE28 (63,5 anos para homens e 64 anos para mulheres).

Quer isto dizer que o crescimento da esperança de vida, em particular das mulheres portuguesas, não tem sido acompanhado de boas condições de saúde. O avançar da idade acarreta diminuição da funcionalidade e aumento da suscetibilidade a doenças. A longevidade humana leva ao aumento de doenças crônicas – cardiovasculares, neoplasias, pulmonares e metabólicas –, devido ao tempo suficiente para se desenvolverem e aos comportamentos de risco, provocadores de incapacidade e morte precoce^2^. Trata-se da perda progressiva de funcionalidade (física e cognitiva), que pode se traduzir em limitações e incapacidade e resultar em dependência.

Percebe-se, portanto, que, quando o prolongamento da vida não é acompanhado de qualidade de vida, os indivíduos são confrontados com necessidades de cuidados e serviços. Esses problemas que o envelhecimento impõe à saúde das populações são demonstrados pelos números relativos à esperança de vida em boa saúde. Apesar de a família ser o principal prestador de cuidados, o envelhecimento demográfico relaciona-se com a própria estrutura e funcionamento das famílias portuguesas. De acordo com o *Eurostat,* em 2017, com uma taxa de fecundidade de 1,38 para Portugal e 1,59 para a UE28, as famílias estão compostas por menor número de pessoas, constituindo uma rede de suporte mais limitada e, portanto, menos capaz de cuidar de seus membros mais velhos. Acrescem ainda fatores como o crescimento do número de uniões de fato, o aumento do número de famílias monoparentais e unipessoais, bem como a redução dos elementos ao nível da família nuclear. Apesar de se verificar aumento do número de famílias trigeracionais, implicando fracas distâncias geracionais, torna-se cada vez menos frequente a coabitação de pais idosos com seus filhos adultos^3^. Lembremo-nos também do aumento de núcleos familiares constituídos apenas por idosos e da alta taxa de mulheres empregadas que Portugal exibe.

Com a diminuição da capacidade das respostas ao nível da família, observa-se um aumento da procura por serviços formais. Estas considerações têm justificado o crescimento do número de estruturas residenciais para pessoas idosas (ERPI) e de serviços de apoio domiciliário (SAD), bem como da Rede Nacional de Cuidados Continuados Integrados (RNCCI). De 2000 a 2015 houve um crescimento de 66% para as ERPI, com taxas de ocupação acima dos 90%^4^. Falamos da internação procurada por questões de incapacidade e como consequência de transição epidemiológica, aumentando as doenças crônicas e incapacitantes. Já a valência SAD, apesar de ter sido a resposta social que mais cresceu nos últimos anos (71% de crescimento entre 2000 e 2015), apresentou a maior queda de taxa de utilização^4^. Pode, assim, denotar algum desfasamento entre as necessidades de indivíduos/famílias e os serviços prestados no domicílio, que poderiam evitar internação e hospitalização prolongada.

Os desafios ao nível da saúde e funcionalidade dos indivíduos mais velhos podem ser analisados ao se refletir sobre os principais motivos que levam a pessoa a ser internada: (i) crescente deterioração física; (ii) incapacidade de os cuidadores informais prestarem os cuidados; (iii) falta de apoios nos serviços domiciliários^5^. Note-se que, de acordo com o *Eurostat,* em 2011, 12,6% das pessoas com 85 anos ou mais viviam em agregados familiares, sendo que apenas 14,8% das mulheres e 7,6% dos homens nessas condições estavam internados. Esta questão torna-se relevante perante o desejo das pessoas permanecerem independentes em suas habitações, em detrimento de qualquer modelo de residência assistida^6^^,^^7^. A maioria das pessoas deseja ficar o maior tempo possível nas próprias habitações e acredita que a casa onde se encontra é onde viverá^8^. Isso porque, na verdade, desejam permanecer num ambiente que lhes seja familiar, em sua casa e comunidade^9^.

Como alternativa à internação precoce e/ou indesejada, começa a tomar forma o paradigma de *ageing in place (AiP)*. No essencial, dá forma aos pressupostos que valorizam que as pessoas envelheçam e permaneçam o maior tempo possível em seu próprio *habitat.* Promover as condições para que tenham a oportunidade de permanecer em casa e envelhecer onde desejam considera-se meta fundamental das políticas sociais dirigidas ao envelhecimento. Para atingir esses objetivos, é necessário que a casa acompanhe o processo de envelhecimento da pessoa e que esta seja capaz de adaptar sua vida e suas condições de habitabilidade. Na verdade, envelhecer em casa e seu bairro implica consciencialização e preparação abrangentes, sobretudo em dois níveis: (i) a adaptação do ecossistema das populações ao longo da vida e (ii) estratégias individuais.

Tendo em consideração esses dois níveis, o presente estudo procurou conhecer as estratégias das pessoas para permanecer em suas habitações o maior tempo possível e a forma como mobilizam os recursos que têm à disposição.

### *Ageing in place* (AiP), um paradigma alternativo para o envelhecimento?

Ao longo das últimas décadas temos assistido a paradigmas cada vez mais positivos dirigidos às políticas públicas e à sociedade em geral, capazes de alterar a forma como se encaram as pessoas mais velhas e o processo de envelhecimento. O conceito de AiP é exemplo dessas orientações mais positivas, que significa viver em casa e na comunidade, com segurança e de forma independente, à medida que se envelhece^10^. Surge como suporte à criação de condições para que as pessoas possam permanecer em seu próprio *habitat* o maior tempo possível conforme envelhecem, mesmo que sofram de alguma doença ou declínio, seja ele funcional ou cognitivo. Com efeito, este paradigma idealiza a casa e a comunidade envolvente como locais privilegiados para envelhecer^11^.

De acordo com Pynoos, Caraviello e Cicero^12^, *AiP* é uma política emergente que se centra principalmente na compreensão das alterações ao longo do envelhecimento e no meio em que a pessoa está inserida. Para Martin, Santinha, Rito e Almeida^13^, *AiP* foca na compreensão de mudanças que ocorrem no envelhecimento e no ambiente envolvente, privilegiando a manutenção da pessoa quer em sua própria habitação, quer noutras situações estruturadas na comunidade.

Reconhece-se que, ao envelhecer em suas comunidades, as pessoas permanecem durante mais tempo independentes e autônomas e mantêm suas redes de suporte social ativas. A internação poderá causar-lhes maior e/ou mais acelerada perda de autonomia, pois as atividades de vida diária (AVD), como cozinhar e limpar a casa, irão desaparecer. Tomasini e Alves^14^ afirmam que os ambientes institucionais exigem muito pouco das pessoas mais velhas, e estas, ao saírem de suas habitações, muitas vezes perdem suas relações sociais, enfraquecendo os laços sociais. Nesta linha argumentativa, sabe-se também que pessoas mais velhas associam AiP a autonomia e independência, no que respeita à possibilidade de realizarem suas escolhas, acederem aos serviços que entenderem, desfrutarem das relações sociais e sentirem-se seguras^9^.

Esta perspectiva do AiP que se situa para além da habitação está relacionada com a literatura e interesse crescentes da gerontologia ambiental, na medida em que todo o ecossistema, sobretudo o bairro onde se envelhece, pode ter efeito na saúde das pessoas, sendo um ambiente onde as pessoas mais velhas podem ter maior sensibilidade, devido a alterações biológicas ou à longevidade das habitações^15^^,^^16^.

Segundo a OCDE^17^, *AiP* compreende quatro dimensões: (i) a habitação, onde é necessário ter em conta a adaptação, a acessibilidade, a manutenção e alternativas habitacionais; (ii) os serviços integrados, nomeadamente os cuidados integrados, SAD e RNCCI; (iii) os transportes, que dizem respeito à forma de mobilidade das pessoas, podendo ser um elo facilitador ou limitador; e (iv) o bairro/comunidade em que a pessoa está envolvida. À semelhança, a OMS considera que o sucesso de um AiP decorre de uma abordagem complexa de vários níveis de intervenção, destacando: pessoas, lugares, produtos, serviços personalizados e políticas de apoio social^10^. Consideremos ainda as dimensões de AiP identificadas por Iecovich^9^, que se relacionam entre si: física (lugar), social (relações pessoais), emocional e psicológica (relacionada ao sentimento de pertença a um lugar) e cultural (relativa a valores e crenças que as pessoas atribuem aos lugares). Na verdade, além de ser considerado vantajoso, o AiP é valorizado pelas pessoas mais velhas em termos de sentimento de apego ou conexão, e de segurança e familiaridade em relação aos lares e às comunidades, bem como pelo senso de identidade, tanto por independência e autonomia, como pelos relacionamentos e papéis de cuidado nos lugares onde vivem^15^.

Este conceito pode ser analisado segundo o modelo ecológico, que arroga que os padrões de bem-estar, saúde e funcionamento da pessoa estão interligados a influências dos recursos biológicos, comportamentais, sociais, físicos e ambientais que circundam a própria pessoa, suas famílias e comunidade^13^. O modelo ecológico, ao ilustrar as mudanças fisiológicas, comportamentais, sociais e ambientais que ocorrem em nível individual e da comunidade, bem como as relações entre essas mudanças que exercem seu papel ao longo do processo de envelhecimento^18^, facilitará a identificação de lacunas, prioridades, necessidades e intervenções.

Wang et al.^19^ consideram que os meios efetivos de apoiar os mais velhos são de importância central na saúde pública, capazes de prolongar uma vida independente. O exercício deste arquétipo, suportado pela promoção da saúde e pela prevenção da dependência ao longo do ciclo de vida, é capaz de produzir efeitos significativos. Isso porque os declínios (físicos e cognitivos) que podem ocorrer com a idade constituem barreiras a um estilo de vida independente, e o incentivo a mudanças comportamentais pode reduzir os gastos em saúde com os mais velhos^20^.

## MÉTODOS

Considerando as possibilidades do AiP, mas também os desafios e dimensões para as pessoas que envelhecem, este itinerário que percorremos teve por objetivo identificar as estratégias desenvolvidas por pessoas em processo de envelhecimento para se manter residindo na própria casa, apesar das fragilidades e dificuldades com que deparam.

A operacionalização do objetivo desta pesquisa implicou a seleção de um município português: Portimão. Segundo os dados do INE - Portugal, neste concelho algarvio, em 2018, 19,8% da população tinha 65 ou mais anos, sendo relativamente inferior aos 21,7% de Portugal como um todo. Ainda assim, é um município bastante envelhecido, com 120,3 idosos por 100 jovens e um 31,1 idosos por 100 pessoas em idade ativa.

Na coleta de dados, realizada em 2017, selecionou-se como técnica a entrevista semiestruturada, com o auxílio de um questionário construído para isso, contendo questões de reposta aberta (curta) e fechada, considerando-se a importância de caracterizar os respondentes do ponto de vista de sua saúde e condições de vida, identificar as necessidades diárias, estratégias utilizadas e aspirações e angústias quando se projetam no futuro.

A amostra foi constituída por residentes do concelho de Portimão, com 65 e mais anos, não internados e que viviam em diferentes condições sociais e de saúde. Os inquiridos foram selecionados por conveniência, sendo pessoas que se disponibilizaram para colaborar com a pesquisa, por uma metodologia de bola de neve, procurando reunir ambos os sexos. No total, foram realizadas 50 entrevistas com pessoas que cumpriram os critérios de inclusão.

Os dados reunidos a partir das entrevistas foram tratados de duas formas diferentes. Em relação às questões fechadas (abordagem quantitativa), foi utilizado o programa de análise estatística IBM SPSS Statistics 25, permitindo a análise estatística uni e bivariada. As questões abertas (abordagem qualitativa) foram submetidas à técnica de análise de conteúdo, possibilitando a construção de categorias.

## RESULTADOS E DISCUSSÃO

### Caracterização da Amostra

A amostra reuniu 50 pessoas, com idades entre 65 e 91 anos, apresentando uma distribuição por várias classes etárias: 46% da amostra tem entre 65 e 75 anos; 42%, entre 75 e 84 anos; e 12%, idade igual ou superior a 85 anos. Em relação ao gênero, a amostra alcançou um bom equilíbrio, com 25 pessoas do gênero feminino e 25 do gênero masculino.

O estado civil que mais prevaleceu foi o de casado, correspondendo a 62% dos inquiridos, sendo 20% viúvos, 14% divorciados e 4% solteiros. A esmagadora maioria da amostra encontrava-se aposentada (92%) e não mantinha nenhuma atividade.

Quanto à escolaridade da amostra, a maioria era pouco escolarizada (50% completaram o primeiro ciclo, 10% o segundo ciclo, e 4% o terceiro ciclo); apenas 8% completaram o ensino secundário, e 12% tinham estudos superiores. Acrescenta-se que 16% dos inquiridos não tinham completado nenhum grau de escolaridade e que os indivíduos com nível de instrução inferior são sobretudo mulheres (68% das pessoas do gênero feminino completaram apenas o primeiro ciclo, e 20% não estudaram). Essa baixa escolaridade vai ao encontro de outros estudos em Portugal com pessoas de 65 e mais anos^21^^–^^23^, devendo ser considerada perante a necessidade de informação e ativação de recursos necessários a um envelhecimento saudável e AiP.

### Condições Habitacionais

A grande maioria das pessoas inquiridas tem casa própria (92%), metade vive numa moradia, a maioria (56%) tem um jardim ou quintal e 82% tem mais de quatro cômodos. A maior parte dos entrevistados está satisfeita com sua habitação em termos de segurança (74% satisfeitos, 18% muito satisfeitos e 2% completamente satisfeitos) e efetua uma avaliação positiva do estado de conservação (60% bom e 12% muito bom). Refletir sobre as condições e melhorias das casas onde se envelhece é fundamental para a análise da saúde das populações, uma vez que a casa pode expor as pessoas a diversos riscos para a saúde. Segundo a OMS^24^, melhorar as condições habitacionais pode salvar vidas, prevenir situações de doença, melhorar a qualidade de vida, reduzir a pobreza e mitigar os efeitos das alterações climáticas. Em relação às modificações que fariam na habitação, destacam-se questões de conforto, como pintura e decoração, e de acessibilidade, como a adequação dos banheiros, por exemplo (Figura 1). Este cenário vai ao encontro da OCDE^25^, que afirma que, em Portugal, viver em habitações com condições satisfatórias é um dos aspectos mais importantes da vida das pessoas. Salienta-se ainda que a grande maioria dos inquiridos (96%), sobretudo os homens (100%), considera que a casa reúne as condições necessárias para habitar em segurança durante mais anos, embora refiram a necessidade de pequenas intervenções quanto ao estado de conservação.

**Figura 1:** Alterações na habitação.

Estes resultados evidenciam o desejo de permanecer o maior tempo possível na própria habitação, colocando a internação como último recurso. Apenas um caso (homem) revelou a opção contrária. No entanto, surgem vários cenários que as pessoas têm consciência de que, se ocorrerem, poderão levar à situação indesejada de deixar sua habitação. Foram referidos problemas de saúde, falta de condições e de segurança da própria habitação e, como expoente máximo, a morte (Figura 2).

**Figura 2:** O que o levaria a sair de sua habitação?

Inquiridas sobre que opções tomariam se saíssem de sua habitação, 24% (12 indivíduos) recorreriam a uma residência coletiva (Figura 3).

**Figura 3:** Opções a tomar.

Sobre as estratégias que utilizam para permanecer mais tempo na própria habitação, as respostas passam por: ser ativo (42%, 21 inquiridos), efetuar alterações na estrutura da habitação (14%, 7), ou ter ajuda de familiares/empregados (6%, 4) (Figura 4). Apesar de inativos, os entrevistados revelaram consciência da importância de estilos de vida saudáveis, de condições de habitação e da prestação de cuidados, seja informal ou formal.

**Figura 4:** Estratégias para permanecer na própria habitação.

### Condições e Gestão da Saúde/Doença

O avançar da idade impõe uma tendência de declínio na saúde. Neste determinante do processo de envelhecimento, muitos inquiridos descrevem sua saúde como razoável (46%), 30% como boa e 16% como má, destacando-se que os mais velhos fazem uma avaliação menos positiva (50% dos indivíduos com 85 ou mais anos consideram ruim sua saúde). Esses resultados menos favoráveis são o resultado de doenças e limitações diagnosticadas.

As patologias mais comuns foram problemas de coluna, hipertensão, diabetes, artroses, problemas respiratórios e problemas do coração. Estes dados vão ao encontro de outro estudo^26^, que conclui que as doenças mais frequentes na população portuguesa com mais de 50 anos consistem em: diabetes, hipertensão, doença reumática, osteoporose, litíase renal e depressão, destacando-se a hipertensão arterial como a condição crônica mais comum.

Apesar das doenças e limitações, bem como de toda a variedade de medicação que os inquiridos afirmam tomar, a maioria nunca sentiu que a idade o impedisse de realizar as atividades que mais gosta e do dia a dia. Porém, com o avançar da idade, especialmente na classe de 85+ anos, o cenário se inverte, e as pessoas já consideram que a idade as impede muitas vezes de realizar atividades. Importa aqui esclarecer que os inquiridos se referiam à vulnerabilidade e à debilidade do estado de saúde que o avançar da idade acarreta, e não aos efeitos da idade por si só.

No que concerne à dimensão de AiP serviço de saúde, nenhum dos inquiridos recorre a qualquer tipologia de serviço domiciliário. Por sua vez, os valores representados na Carta Social^4^ referentes ao concelho de Portimão revelam que a resposta social do SAD tem capacidade para 295 utentes, estando a ser usufruído apenas por 179. Percebemos, portanto, que os entrevistados revelaram preocupações em torno da prestação de cuidados sociais e de saúde no futuro. Contudo, a relação entre as necessidades e a diversidade de serviços existentes/disponíveis requer maior exploração. Ainda assim, verificamos que esses resultados estão em concordância com a literatura acerca do significado de AiP para as pessoas mais velhas, enfatizando que um AiP de sucesso requer a prestação de serviços de saúde em casa^27^.

### Meio de Transporte Utilizado

Relativamente à dimensão dos transportes, verificou-se que sua utilização varia consoante a idade. Na classe 65-74 anos, o tipo de transporte mais usado é o carro; na classe 75-84 anos, são os transportes públicos; e na classe 85+ anos, os resultados dividem-se entre andar a pé e transportes públicos. Em relação ao gênero, homens utilizam mais o carro próprio, e mulheres, os transportes públicos. Neste campo de intervenção, são sugeridas várias alterações com o intuito de melhorar a rede de transportes, bem como os próprios interesses das pessoas, isto é, trajetos mais diretos e com mais frequência, indicando ainda a existência de transportes aos fins de semana. O fato de a rede de transportes não corresponder às necessidades das pessoas poderá resultar em isolamento e diminuição de interações sociais, caso as pessoas não tenham maneira de se deslocar, acabando muitas vezes por não sair de casa. Estas considerações são cada vez mais destacadas pela literatura de AiP, ao sublinhar que é preciso considerar não apenas as opções de habitação, mas também o transporte, que efetua a ligação das pessoas com as oportunidades recreativas, a interação social, o envolvimento cultural e a educação permanente^15^^,^^28^.

### Características da Comunidade onde se Envelhece

A maioria das pessoas inquiridas sente-se segura e considera que a comunidade dispõe dos recursos necessários para o dia a dia. No entanto, destaca algumas necessidades, como enfermarias, lares, lugares de convívio e um talho, por exemplo. O simples fato de as pessoas se sentirem seguras em sua comunidade influencia as condições de independência, saúde física, integração social e bem-estar emocional, por se movimentarem sem receios^29^. Todas as pessoas referiram uma panóplia de atividades que compõem seu cotidiano, e, no que diz respeito a aspirações e angústias, os inquiridos manifestaram o desejo de permanecer em sua própria habitação e na comunidade, colocando a internação em último plano e indesejada, ainda que seja a única opção para alguns. Pudemos ainda apurar uma preocupação com os problemas de saúde, associados ao aparecimento de doenças, com a incapacidade física, e apreensão em relação à solidão.

Para ter mais qualidade de vida, a amostra considerou que precisa “manter a mesma vida”, continuando a realizar as mesmas atividades ao longo da vida, tendo a capacidade para realizar as AVD e AIVD. Este conjunto de respostas remete para três focos de intervenção necessários à concretização de um AiP: (i) estratégias relacionadas com habitação; (ii) adequação dos serviços de saúde; e (iii) participação e envolvimento ativo ao longo da vida.

## CONCLUSÃO

A pesquisa permitiu identificar preocupações e estratégias que as pessoas desenvolvem para permanecer em suas habitações o maior tempo possível, ao longo do processo de envelhecimento, bem como a forma como mobilizam os recursos que têm à disposição. As condições e necessidades de saúde ao longo da vida assumem destaque quer em termos das percepções das pessoas inquiridas, quer na literatura que suporta os desafios e exigências da prática de AiP. No centro da decisão e possibilidade de AiP estão as condições habitacionais e de habitabilidade. Mas outras variáveis assumem destaque na pesquisa, como as condições econômicas e de saúde e a rede de suporte social. Os documentos estratégicos da OMS^24^ já alertam para a interligação de todas essas dimensões, relacionando as características das casas com os riscos de doença.

No entanto, a concretização do paradigma AiP suporta um leque extenso de necessidades, que podem ser entendidas como constrangimentos. Na amostra estudada, falamos em particular do problema econômico, na medida em que a maioria das pessoas inquiridas revelou baixa literacia e baixos recursos econômicos, podendo limitar a realização de alterações/mudanças necessárias em sua habitação, por exemplo. Estas questões cruzam-se com o AiP e a saúde pública na medida em que existe uma preocupação crescente com a qualidade das habitações das pessoas que envelhecem, em termos de isolamento, aquecimento/arrefecimento, dimensão e *design*/acessibilidades^15^^,^^30^.

Outra dimensão dos constrangimentos do AiP diz respeito a serviços. Ainda que a análise dos cuidados domiciliários se apresente menos dispendiosa que os cuidados institucionais^31^, a disponibilidade dos SAD no contexto português é reduzida, e a produção desses bens, tendencialmente onerosos por parte do mercado existente, gera desigualdades, excluindo parte da população, mais velha e com baixos rendimentos.

Estas conclusões levam-nos ainda a refletir sobre a desigualdade de oportunidades para o envelhecimento na comunidade, isto é: será que para a população com baixos rendimentos o envelhecimento na comunidade pode representar uma má qualidade de vida? Por oposição, pessoas com melhores condições econômicas terão melhor capacidade de adaptação e permanência em suas casas?

A idade e limitações que esta impõe não têm de forçar ou decretar, necessariamente, a mudança para uma residência coletiva (ERPI), principalmente se os indivíduos ainda não padecem de problemas capazes de impedir a vida em comunidade, sejam eles físicos, psicológicos ou sociais. É neste sentido que emergem projetos comunitários e acadêmicos em torno de estratégias para que se permaneça e envelheça em suas habitações. No relatório de Fonseca^32^ são partilhadas 81 iniciativas, sistematizadas por categorias de intervenção: apoio a cuidadores; combate ao isolamento; gerotecnologias e investigação; inovação em apoio domiciliário; inovação em centro de dia; intervenção na vida da comunidade; lazer, atividade física e aprendizagem ao longo da vida; melhoria das condições de habitação; recursos de saúde, animação, nutrição e acompanhamento psicológico; segurança, mobilidade e bem-estar.

Contudo, apesar do desejo evidenciado na pesquisa de envelhecer em seu *habitat*, é importante relembrar que os próprios entrevistados não menosprezam a internação e suas opções, quando necessário. São cenários de fragilidade e doença grave, com a necessidade de cuidados de saúde em tempo integral, ou situações em que a habitação já não se adequa às limitações da funcionalidade. Ainda assim, e mesmo nestas situações, os inquiridos consideram a internação como último recurso, dando prioridade aos familiares, nomeadamente os filhos, afirmando que recorreriam a serviços de cuidados informais para auxílio no dia a dia.

As estratégias e necessidades apontadas pelos entrevistados salientam intervenções em vários níveis, que constituem a conclusão central desta pesquisa. O primeiro nível diz respeito a habitações. Apesar de satisfeitos com suas casas, os inquiridos reconhecem que no futuro será necessário efetuar adaptações para que se mantenham seguras e permitam envelhecer nelas. Num segundo nível encontra-se a prestação de serviços integrados (sociais e de saúde), quer através de apoio domiciliário, quer sob a configuração de serviço permanente, ou seja, de um cuidador mais presente no dia a dia da pessoa. O terceiro setor de intervenção diz respeito à adequação dos serviços da comunidade, que passa pelas características e funcionamento dos transportes públicos, ajustando-os aos interesses da população que envelhece. Isto porque, apesar de se sentirem seguros e mobilizarem de forma positiva os recursos que têm à disposição, as pessoas que vivem mais distantes do centro da cidade sentem falta de dispositivos de proximidade.
